# Association of Salty and Sweet Taste Recognition with Food Reward and Subjective Control of Eating Behavior

**DOI:** 10.3390/nu16162661

**Published:** 2024-08-12

**Authors:** Imke Schamarek, Florian Christoph Richter, Graham Finlayson, Anke Tönjes, Michael Stumvoll, Matthias Blüher, Kerstin Rohde-Zimmermann

**Affiliations:** 1 Medical Department III, Division of Endocrinology, Nephrology, Rheumatology, University of Leipzig Medical Center, 04103 Leipzig, Germany; anke.toenjes@medizin.uni-leipzig.de (A.T.); matthias.blueher@medizin.uni-leipzig.de (M.B.); 2 Helmholtz-Institute for Metabolic, Obesity and Vascular Research (HI-MAG), Helmholtz Center Munich at the University of Leipzig and the University Hospital Leipzig AöR, 04103 Leipzig, Germany; 3 Department of Anesthesiology and Critical Care Medicine, University Hospital Leipzig, 04103 Leipzig, Germany; 4 Appetite Control and Energy Balance Research, School of Psychology, Faculty of Medicine and Health, University of Leeds, Leeds LS2 9JT, UK; g.s.finlayson@leeds.ac.uk

**Keywords:** food reward, liking and wanting, taste, obesity, eating behavior

## Abstract

Sweet and salty tastes are highly palatable and drive food consumption and potentially uncontrolled eating, but it remains unresolved whether the ability to recognize sweet and salty affects food reward and uncontrolled eating. We investigate the association of sweet and salty taste recognition with liking and wanting and uncontrolled eating. Thirty-eight, mainly female (68%) participants of the Obese Taste Bud study, between 22 and 67 years old, with a median BMI of 25.74 kg/m^2^ (interquartile range: 9.78 kg/m^2^) completed a taste test, the Leeds Food Preference Questionnaire to assess food reward, the Power of Food Scale (PFS) and the Three-Factor Eating Questionnaire to assess different aspects of uncontrolled eating. Better salty taste recognition predicted greater implicit wanting for high-fat savory foods (β = 0.428, *p* = 0.008) and higher PFS total (β = 0.315; *p* = 0.004) and PFS present subscale scores (β = 0.494, *p* = 0.002). While neither sweet nor salty taste recognition differed between lean individuals and individuals with obesity, those with greater trait uncontrolled eating showed significantly better salty taste recognition (U = 249.0; *p* = 0.009). Sweet taste recognition did not associate with food reward or uncontrolled eating. Better salty but not sweet taste recognition associates with a greater motivation for, but not liking of, particularly savory high-fat foods and further relates to greater loss of control over eating. Salty taste perception, with taste recognition in particular, may comprise a target to modulate food reward and uncontrolled eating.

## 1. Introduction

The taste of food is a potent driver for food consumption [1,2]. From an evolutionary perspective, how a certain food tastes provides the consumer with information on the nutritional benefit of their meal, promoting health and survival [3]. For instance, a sweet taste usually signals calorie-dense food and low risk for harm [3]. A salty taste contributes to an optimal sodium balance and consumption is stimulated or inhibited depending on homeostatic needs which is closely tied to fluid balance [4,5,6].

In the modern, industrialized world, taste has increasingly lost its purpose to consume foods according to homeostatic needs but rather serves hedonic need fulfillment; we no longer eat what we need, but what we experience as palatable [1,7,8].

Particularly, sweet and salty tastes are used in hyper-palatable foods that target reward and often trigger uncontrolled eating [9,10]. In an environment where food is constantly available, food reward and uncontrolled eating may contribute to overconsumption and ultimately to obesity development [11]. While foods are undoubtedly designed to taste good, people show differences in their ability to recognize taste [12]. But do differences in sweet and salty taste recognition, as one aspect of taste perception, associate with hedonic, reward seeking or uncontrolled eating behavior?

Most research associating taste perception with food reward and uncontrolled eating behavior was conducted on individuals with obesity, investigating the hypothesis that changes in particularly sweet taste perception impact food reward and uncontrolled eating behavior and consequently drive food selection and intake [13]. However, to date, these proposed connections remain insufficiently resolved. First, findings regarding taste perception in obesity are diverging and range from being reduced to increased or not different from normal-weight individuals [13,14,15]. One aspect contributing to varying results is methodological heterogeneity in studies of taste perception in obesity. Taste perception often refers to very different dimensions of taste (e.g. intensity, recognition or pleasantness) which, nevertheless, are often subsumed under the term ‘taste perception’ [13].

Secondly, the association between taste perception and food reward or uncontrolled eating is usually addressed by self-reported food liking and food frequency questionnaires or in disordered binge eating, supposedly reflecting uncontrolled eating [2,15]. Not only do these studies yield inconclusive results, they also do not assess all relevant dimensions of food reward and ignore that uncontrolled eating in disordered eating may not be representative for a healthy population.

When investigating food reward, it is important to acknowledge its dual concept distinguishing liking and wanting which can operate with and without explicit awareness [16]. Liking describes an affective component which can be consciously described by an individual as subjective pleasure derived from tasting a certain food (explicit liking). This dimension is most often employed when referring to “food reward”. Wanting is a motivational component of (food) reward, which expresses itself as a conscious desire to consume a specific food (explicit wanting), while the implicit counterpart is the drive to eat triggered by a food cue following processes of incentive salience attribution (implicit wanting) [16,17]. As particularly implicit wanting is more complex to assess, it is often neglected when assessing food reward, yielding an incomplete investigation of the construct.

Uncontrolled eating in the general population is the result of a strong food reward signal, also referred to as hedonic hunger, in relation to poor ability to exert inhibitory control [18]. Hedonic hunger describes one’s preoccupation with and desire to consume foods for the mere purpose to seek pleasure although not physically hungry, triggered by implicit or explicit awareness of palatable foods in their environment which is reflected by feelings of being controlled by food independent of caloric needs [19].

Interestingly, dimensions of salty taste perception, despite their relevance in food palatability, have been widely neglected in the context of food reward and uncontrolled eating behavior. One reason might be that while the physiology of sweet taste perception is well understood, the exact mechanisms of salty taste perception remain under debate [14,20,21]. Briefly, sweet taste is mainly sensed by the heterodimeric T1R2/T1R3 G-protein-coupled receptors located on type II taste bud cells mostly found on the tongue’s surface. Tastants bind to these receptors and information on taste is further transmitted to and processed in the primary gustatory cortex. Salty taste perception commences with epithelial Na channels (ENaC) and transient receptor potential cation channel subfamily V member 1 (TRPV1) [20]. It is assumed that the first is responsible for appetitive responses to low salt concentrations and the latter for aversive responses to high salt concentrations [20]. This dual form of processing is thought to be of relevance in a usually observed inverted U-function that describes the association between saltiness and palatability which further complicates investigations regarding dimensions of salty taste perception and food reward [22].

However, that processes involved in salty taste perception indeed relate to reward experience and potentially uncontrolled eating is suggested by brain imaging studies which show that sensory salty taste processing is accomplished by brain circuits also involved in reward, executive control and, particularly, control over eating [23,24,25]. Studies in animals and humans have shown that salt appetite is increased during salt-depleted situations [6]. Salt appetite describes the motivation to consume dietary salt and its rewarding palatability when tasted. This is driven by several highly conserved but distinctive neuronal pathways that integrate information related to homeostatic salt status, the detection of cues and gustatory salt input [6].

In sum, whether taste perception, and more precisely, an individual’s ability to recognize sweet and salty tastes, relates to food reward and uncontrolled eating remains unknown and the nature of these potential associations and their possible implication in weight regulation need further investigation. Given that other taste qualities, such as sour and bitter tastes, are of less relevance in the context of palatability and that it is recurrently debated whether umami truly comprises a basic taste quality, the present work focuses on sweet and salty taste recognition only [26,27].

Therefore, the aim of the present study is the exploration of potential associations of both, sweet and salty taste recognition with food reward and uncontrolled eating behavior in lean individuals and individuals with obesity. To overcome previous limitations, we assess both, liking and wanting as two distinguishable dimensions of food reward and include different measures to assess uncontrolled eating in a population without eating disorders. A secondary aim is to further explore the association of other eating styles (restrained eating, emotional eating, disinhibition, cognitive restraint and hunger) commonly associated with overconsumption with salty and sweet taste recognition.

## 2. Methods

### 2.1. Recruitment and Study Population

Participants were recruited as part of the observational, prospective “Obese Taste Bud” (OTB) Study (Clinicaltrail.gov: NCT04633109 or German Registry for Clinical Studies: DRKS00022950) from the general population [28]. The study population is described in greater detail elsewhere [28]. Participants were recruited through advertisement on the institutional homepage and through flyers within the campus of the University Hospital of Leipzig. Potential participants were screened for eligibility in a telephone interview, during which a trained research assistant assessed inclusion and exclusion criteria: male and female participants had to be aged between 18 and 69 years and were not included when suffering from any severe kidney, heart, liver, neurological or mental disease, including eating disorders; were diagnosed with a malignant disease; or had experienced radiation, chemotherapy or recent surgery. Participants were further excluded if suffering from any disorder that can directly impact taste perception or if current substance abuse, steroid use, pregnancy or breast feeding was reported. The present analyses were conducted in a subsample of the original OTB cohort, as complete data were only available for 38 participants. In more detail, the German version of the Leeds Food Preference Questionnaire (LFPQ-G) was completed by 61 participants, since the instrument’s development and validation were completed after recruitment for the OTB study had started already. Data of the LFPQ-G were incomplete in nine participants, which were excluded in the present analyses. One participant had to be excluded due to a BMI below <18.5 kg/m^2^ which was defined as an exclusion criterion but had not been noticed during recruitment. In seven participants, data on taste perception were missing, and in six participants, questionnaire-based data on relevant variables were not available.

### 2.2. Study Design

Participants arrived at the outpatient unit of the University Hospital of Leipzig or the Helmholtz-Institute for Metabolic, Obesity and Vascular Research (HI-MAG) at 7:30 o’clock in the morning after overnight fasting (>12 h). Participants were further asked to refrain from brushing their teeth for 45 min or chewing gum or smoking 30 min prior to their visit. Upon arrival, informed written consent was obtained. All procedures meet the standards of the Declaration of Helsinki and were approved by the ethics committee of Leipzig University, Leipzig. Informed written consent was obtained from all participants. The study is described in greater detail elsewhere [28]. Participants’ characteristics, medical and behavioral data were assessed during a standardized interview and through self-reports via questionnaires. As investigating taste perception and its implications in eating behavior and obesity is one of the original aims of the OTB study, numerous aspects related to taste were assessed, including taste recognition and taste liking. The present analyses focus on taste recognition only.

### 2.3. Taste Recognition

Commercially available taste strips (Burckhart Odofin taste strips, Medisense, Germany) were used to assess taste recognition. Taste strips are filter papers impregnated with different solutions creating each of the four basic tastes, namely sweet (sucrose), sour (citric acid), salty (sodium chloride) and bitter (quinine hydrochloride). Taste strips were placed in the middle of the participant’s tongue, who was then asked to identify the presented taste. Eighteen taste strips were presented according to a standardized protocol during which each taste quality is presented in four increasing concentrations in a randomized order. Two non-impregnated control strips are included, which are not considered in the final taste score. Participants evaluated each presented taste strip on a computer via LimeSurvey [29]. After each trial, participants were asked to rinse their mouth with water, which was spat out into a container and was not swallowed. Subscales for each taste quality were calculated based on the number of correctly identified taste strips varying from 0 to 4. A sum score was calculated as the sum of all correctly identified taste strips (0 to 16). For the present analyses, only salty and sweet taste recognition were considered.

### 2.4. Assessment of Food Reward and Control over Eating Behavior

The following instruments were employed to assess all relevant components of food reward and uncontrolled eating:

#### 2.4.1. The Leeds Food Preference Questionnaire

The German version of the Leeds Food Preference Questionnaire (LFPQ-G) was used to assess food reward [30]. The LFPQ was originally developed in the UK by Finlayson et al. (2007) [31]. The original and the German instrument underwent a standardized validation process which is reported in greater detail elsewhere [30,32]. The LFPQ-G is a computerized instrument which assesses the distinguishable dimensions of liking and wanting separately for different food categories varying in taste (sweet vs. savory) and fat content (high vs. low fat). Four food images representing each of the four food categories, high-fat savory (HFSA), low-fat savory (LFSA), high-fat sweet (HFSW) and low-fat sweet, (LFSW) are presented individually to assess explicit liking (“How pleasant would it be to taste some of this food now”?) and explicit wanting (“How much do you want some of this food now?”). Food images are presented pairwise in a forced choice paradigm in which participants are asked to react as quickly as possible to the question “Which of the presented foods do you most want to eat now”? to assess implicit wanting of a certain food category. Implicit wanting is calculated according to a specific algorithm that includes the number of positive and negative reactions to a particular food image as well as reaction time.

#### 2.4.2. Power of Food Scale

The Power of Food Scale (PFS) assesses individual differences in hedonic hunger [33]. The PFS consists of 15 items which are answered on a 5-point Likert scale anchored with “I don’t agree at all (=0)” and “I strongly agree (=5)”. Three different subscales further differentiate the proximity of food to people necessary to trigger appetitive motivation. ‘Food availability’ assesses the preoccupation, and hence appetitive motivation, when food is not directly present but constantly available. ‘Food present’ assesses appetitive motivation when food is present, and ‘food taste’ assesses the drive to eat for pleasure when food has commenced the process of tasting. The German PFS demonstrates good internal consistency for the total scale (α = 0.92). [34] Of note, the PFS does not contain items reflecting quantity or frequency of actual palatable food consumed, which allows us to distinguish between actual consumption vs. motivation to consume [19]. Higher scores signal greater appetitive motivation and less subjective control in response to food-abundant environments.

#### 2.4.3. Three-Factor Eating Questionnaire

The German version of the 51-item Three-Factor Eating Questionnaire (TFEQ) was applied to assess characteristics of participants’ eating behavior [35]. All items are coded with 0 or 1. Scoring followed the same procedure as proposed in the original instrument, forming the subscales ‘cognitive restraint’, ‘disinhibition’ and ‘hunger’ [36]. We further included the subscales based on the factor structure identified in a large validation study of the German version of the TFEQ: ‘uncontrolled eating’ contains 11 items of the original hunger and disinhibition factors. ‘Emotional eating’ contains three items of the original disinhibition factor which assesses eating caused by emotional triggers like feeling anxious or depressed [35]. ‘Cognitive restraint’ contains 15 restraint items of the original TFEQ. Cronbach’s alpha for the factors ‘restraint eating’, ‘uncontrolled eating’ and ‘emotional eating’ were 0.840, 0.802 and 0.780, respectively [35]. Therefore, the instrument shows good internal consistency.

### 2.5. Anthropometric Measures

Body weight and height were measured on a calibrated scale with stadiometer (Seca, Hamburg, Germany) without shoes to the nearest of 0.5 kg and 0.1 cm, respectively. Body mass index (BMI) was calculated as body weight (kg) divided by body height squared (m^2^).

### 2.6. Statistical Analysis

Data were analyzed using SPSS Version 29 (IBM, Ehningen, Germany) and GraphPad Prism Version 10. Descriptive data were reported as median, 25th and 75th percentiles. The Mann–Whitney U–test was used to test for group differences between lean participants and participants with obesity, and individuals with high and low TFEQ—uncontrolled eating. The chi-squared test was applied to test for group differences of categorial variables. Participants were grouped into obese and normal–weight subgroups according to their BMI, applying a cut-off of 30 kg/m^2,^ following the definition of the WHO, and a median split was performed to differentiate between high and low uncontrolled eating. Spearman’s rank correlation coefficient was applied to analyze associations between taste perception and LFPQ-G outcome measures, PFS scores and TFEQ—eating behavior, as well as other participants’ characteristics as potential confounding variables. Multiple linear regression analysis was conducted to test whether taste perception predicts relevant outcome measures, controlling for potential confounders, based on previous correlation analysis (Appendix A). An alpha level of <0.05 was considered statistically significant.

## 3. Results

### 3.1. Study Population

**Figure 1:**
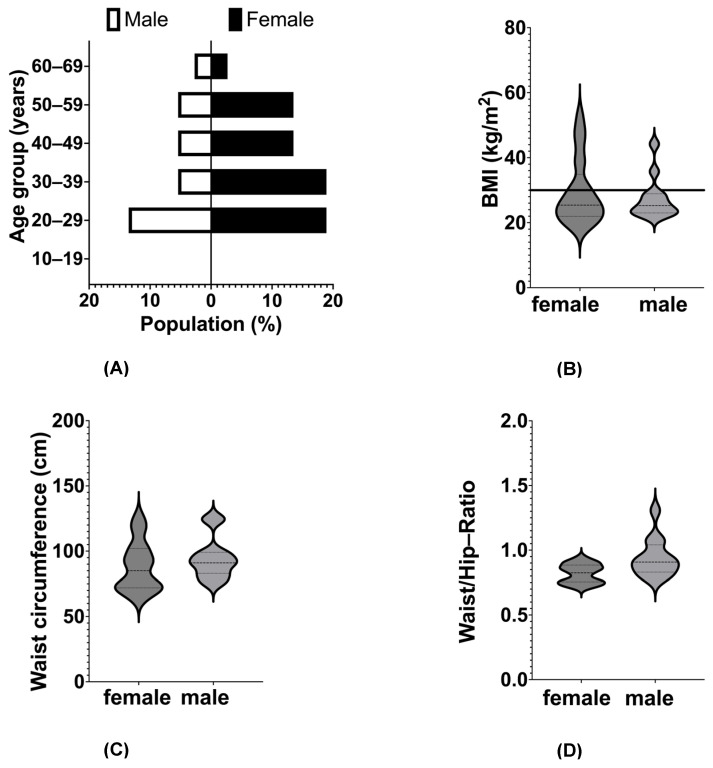
Main characteristics of the study population (N = 38) stratified by sex. Dotted lines indicate 25th and 75th percentiles, dashed lines indicate subgroup’s median and the solid line indicates the cut-off for BMI of 30 kg/m^2^. (**A**) Population pyramid for age. (**B**) Distribution of BMI. (**C**) Distribution of waist circumference. (**D**) Distribution of waist-to-hip-ratio. Abbreviations: BMI, body mass index.

The participants included in these analyses were mostly female and aged between 22 and 67 years. The main characteristics of the study population are demonstrated in greater detail in Table 1, stratified by weight status, and Figure 1, stratified by sex. As the aim of the OTB study is to investigate differences in taste perception and taste cell homeostasis in a general population of lean people and individuals with obesity, the present sample covers a wide range of BMI and age, although the majority is normal -weight with less abdominal obesity, as indicated by waist circumference and waist-to-hip-ratio (Figure 1). As expected, normal-weight participants and those with obesity showed significant differences in BMI and body weight (Table 1). Furthermore, obese individuals smoked significantly more often and scored higher in trait disinhibition (Table 1). However, no differences became evident with regard to taste perception, food reward or variables operationalizing different aspects of uncontrolled eating.

### 3.2. Association of Sweet and Salty Taste Recognition with Liking and Wanting (Food Reward)

Salty taste recognition associated significantly with implicit wanting for HFSA foods. Sweet taste recognition inversely correlated with implicit wanting for LFSA foods (Table 2) but did not remain significant when controlling for age as a potential confounding variable identified in previous analyses (Appendix A). A multiple linear regression analysis was used to test if salty taste recognition significantly predicts implicit wanting for HFSA foods when including smoking as an additional predictor into a final model. Smoking was identified as an additional independent variable based on previous correlation analysis (Appendix A). The overall regression was statistically significant (R^2^ = 0.186, F_(3,34)_ = 3.82, *p* = 0.018). It was found that the association between salty taste recognition remained significant after adjusting for smoking and significantly predicted implicit wanting for HFSA foods (β = 0.428, *p* = 0.008). Sweet and salty taste recognition did not associate with explicit liking or explicit wanting (Table 2).

### 3.3. Association of Sweet and Salty Taste Recognition with Measures of Uncontrolled Eating

Salty taste recognition was positively associated with the PFS total score and the PFS present subscale (Table 3). Sweet taste recognition also associated positively with the PFS total score and the PFS taste subscale (Table 3). However, the latter association did not remain significant after controlling for age. A multiple linear regression analysis was used to test if salty taste recognition significantly predicts PFS total scores when including age as an additional predictor in a final model, as age was associated with PFS total scores as presented in Appendix A. The overall regression for the PFS total score, including salty taste recognition and age in the final model, was statistically significant (R^2^ = 0.268, F_(4,33)_ = 7.59, *p* = 0.002). It was found that in the final model, salty taste recognition significantly predicted scores of the PFS total score (β = 0.315, *p* = 0.004). Furthermore, the overall regression for the PFS present subscale was statistically significant (R^2^ = 0.251, F_(4,33)_ = 7.21, *p* = 0.002), including salty taste recognition and age in the final model. It was found that salty taste recognition significantly predicted scores of the PFS present subscale in the final model (β = 0.494, *p* = 0.002).

**Figure 2:**
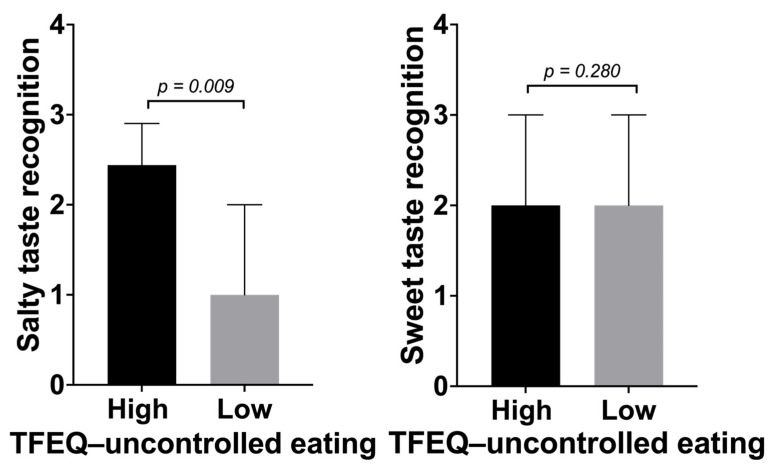
Uncontrolled eating and sweet and salty taste recognition. High vs. low uncontrolled eating was defined according to a median split. Individuals that score high in TFEQ—uncontrolled eating showed significantly higher salty taste recognition but not sweet taste recognition compared to individuals with low scores in TFEQ—uncontrolled eating after applying a Mann–Whitney U-test (GraphPad Prism, Version 10). Abbreviations: TFEQ, Three-Factor Eating Questionnaire.

As presented in Figure 2, group comparison showed that those scoring high in TFEQ—uncontrolled eating showed significantly better salty taste recognition than those scoring low (U = 249,0; *p* = 0.009). Further exploratory analyses showed that neither salty nor sweet taste recognition associated with any of the other eating styles assessed with the TFEQ.

### 3.4. Taste Recognition and Obesity

**Figure 3:**
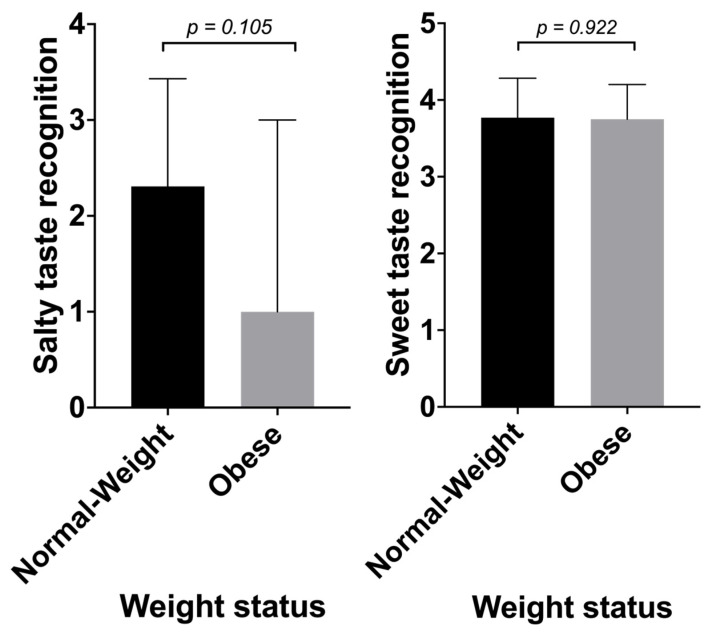
Differences in weight status and sweet and salty taste recognition. Obese and normal-weight individuals were defined according to BMI with an applied cut-off at 30 kg/m^2^ (N = 12 obese and N = 26 normal-weight). Obese vs. normal-weight individuals did not show significant differences in either sweet or salty taste recognition (*p* > 0.05), after applying a Mann–Whitney U–test. (GraphPad Prism, Version 10).

Neither sweet nor salty taste recognition associated with BMI. A comparison of obese vs. normal-weight individuals did not show significant differences between these two groups regarding their ability to recognize sweet or salty tastes (Figure 3). Adjusting for age when investigating sweet taste recognition did not change the results.

## 4. Discussion

The present study explores the association of salty and sweet taste recognition with food reward as well as uncontrolled eating and potential consequences for weight regulation. Results suggest that better salty taste recognition might be associated with greater motivation for, but not liking of, particularly savory high-fat foods and, moreover, appears to relate to greater loss of control over eating, which, however, does not seem to affect weight status.

### 4.1. Association of Salty Taste Recognition with Food Reward and Uncontrolled Eating

Results suggest that better salty taste recognition predicts greater implicit wanting for specifically savory high-fat foods, while not associated to explicit or implicit liking for any food category. These results not only suggest an implication of salty taste recognition regarding food reward, but also show for the first time that better salty taste recognition appears to relate to subconscious motivation following processes of incentive salience rather than affecting conscious liking or wanting for food. Importantly, this association appears to be highly specific to savory high-fat foods. Furthermore, individuals with better salty recognition seem to experience greater awareness of and motivation for palatable foods in their environment when not hungry, as assessed by the PFS. In extreme cases, this could be perceived as a loss of control over food consumption in a food-abundant environment. This assumption is supported by the finding that people who scored higher in measures of trait uncontrolled eating showed significant better salty taste recognition than individuals with a lower tendency for uncontrolled eating.

In line with previous findings, other investigated trait eating behaviors related to overconsumption were independent of salty or sweet taste recognition [15]. This may indicate that the observed connection of salty taste recognition and uncontrolled eating could be unique and rather specific.

A speculated reason for a potential role of specifically salty taste recognition in motivational components of food reward and uncontrolled eating might be the exclusive role of sodium in body fluid balance, in which salt appetite has been identified as a driver for salt consumption depending on homeostatic needs [4,5,6]. In that homeostatic mindset, better salty taste recognition may facilitate the detection of salty foods in the environment and a close connection between salty taste perception, also involving salty taste recognition, and experiencing food reward may increase the likelihood of salt intake when needed [5,6].

We appreciate from animal studies that the behavioral expression of salt appetite is mediated by brain regions that affect its motivational but not hedonic response, and beyond that, the wanting and liking of salt have been shown to be processed by separate brain circuits [25]. Separable pathways might be one reason why better salty taste recognition may not necessarily result in the greater liking of salty foods but in an increased drive for a food category that has been experienced as salty-tasting before. Applied to the modern food environment, results may raise the assumption that individuals with better salty taste recognition may be prone to unhealthy food selection through the effects of better salty taste recognition and an increased implicit drive to consume a specific food category that best meets the desired taste quality. However, these assumptions remain speculative, as brain imaging studies have not been applied in the present study and behavioral data were mainly conducted based on questionnaires. Additionally, previous studies show a lack of evidence connecting salty taste perception with food intake [37].

The specific increase in implicit wanting for savory high-fat but not savory low-fat foods could potentially be related to previous experience of salt and fat being commonly combined taste features increasingly present in processed foods [10]. However, this assumption is speculative. Furthermore, the LFPQ-G low-fat food category comprised some non-salty items like vegetables [30]. However, it is worth noticing that food items within the savory low-fat category also comprised bread, which is considered a sodium-rich food [38]. Also possible are further motivational aspects that particularly connect with fat taste [39].

Assuming that salty taste perception might be implicated in regulating sodium intake, a greater ability to recognize salty taste could facilitate the detection of salty foods in the environment [40]. This in turn may contribute to implicit or explicit awareness of food in the environment by processes that potentially operate independently of hunger. As salty foods compose a large part of hyper-palatable foods, better salty taste recognition, potentially entailed by an increased awareness of salty foods in the environment, may constantly challenge executive functioning as food reward may be overly addressed. This could potentially result in the subjective feeling of being controlled by that food. It might be speculated that the association between better salty taste recognition and greater trait uncontrolled eating may reflect this constant challenge of executive function.

To our knowledge, only a single study addressed the association of human taste perception with uncontrolled eating thought to be a major characteristic in a population of individuals with binge eating disorder [2]. Contrasting our results, individuals with obesity and binge eating disorder were less able to detect salt compared to a control group and individuals with obesity but without binge eating disorder [2]. However, the results were conducted in a sample with diagnosed eating disorders which can entail profound neurobiological changes with disturbed executive functioning or even peripheral taste function. Also, uncontrolled eating was not directly assessed, but assumed present in diagnosed binge eating disorder.

### 4.2. Association of Sweet Taste Recognition and Food Reward and Uncontrolled Eating

In strong contrast to salty taste recognition, in the present study, sweet taste recognition did not associate with any component of food reward, hedonic hunger or uncontrolled eating. To date, the association between different dimensions of sweet taste perception and reward or uncontrolled eating often remains speculative [13]. Most studies investigated the association between sweet taste perception and sweet food intake, both acutely or habitually, assuming an increased food reward or reduced control over eating without actual assessment of these constructs [2,13]. Only very few studies directly addressed the connection of sweet taste perception and food reward, all of which used brain imaging to operationalize food reward and support a positive association between sweet taste perception and food reward [41]. These results contrast the findings of the present study, which, however, did not use brain imaging but focused on different questionnaire-based measures of food reward and uncontrolled eating, which, nevertheless, might mirror actual behavior more closely than mere brain activation does. Interestingly, one study demonstrated a greater activation of reward-associated brain regions to salt compared to sucrose [42]. In line with the aforementioned assumptions the authors argued that the higher sensitivity of these brain regions to sodium concentration may reflect the need for stricter monitoring of sodium intake than sucrose intake given the far more severe consequences of disturbed sodium and fluid balance, while sugar overconsumption is more tolerable [42]. These originally homeostatic mechanisms which connect salty taste more closely with reward and control than sweet taste may account for the observed pattern in that and the present study, according to which different dimensions of salty taste perception relate more closely to food reward and uncontrolled eating than aspects of sweet taste perception.

### 4.3. Taste Recognition and Obesity

The present study sample shows no differences in sweet or salty taste recognition between lean individuals and individuals with obesity. Previous studies show largely diverging results which is in part a consequence of variations in the assessment of taste perception and the study designs applied [2,13,14,43]. Nevertheless, varying outcomes may also indicate larger inter- and intraindividual variation in taste perception, including taste recognition, than assumed, which in turn suggest that a multitude of factors influence taste recognition. Indeed, habitual food consumption, state of hunger, mood, physical activity and even sound have been shown to impact taste perception [44,45,46,47,48]. Present results support the notion that individual taste perception, including taste recognition, is regulated by more complex mechanisms which may not correspond to an individual’s weight status per se [13]. Previously reported differences may reflect distinct phenotype differences associated with obesity. This could include metabolic disease, which was not dominant in the present sample.

### 4.4. Strength and Limitations

This study shows some limitations but is also characterized by multiple strengths. A limitation arises from the cross-sectional design of the study which does not allow us to draw causal conclusions. Longitudinal and interventional studies are needed to further investigate whether better salty taste recognition increases motivation for particularly savory high-fat foods and subjective feelings of uncontrolled eating. In addition, analyses were not corrected for multiple testing; therefore, results have to be interpreted with caution. Furthermore, the correlation coefficient of most of the here presented results is between −0.5 and +0.5, which indicates only moderate strong effects. Therefore, overestimation has to be avoided when interpreting the data. Furthermore, the potential effect of current salt depletion or current hunger was not assessed [44,45,49]. However, no variables of interest were associated with current hunger ratings. Moreover, the potentially implicated neurological mechanisms discussed here remain speculative as brain imaging studies were not part of the study. Beyond that, the relevance for actual food consumption remains unclear, as the latter was not assessed. A strength is the differentiation between affective and motivational food reward dimensions using the LFPQ-G. Furthermore, a sample of healthy individuals without diagnosed eating disorders allows generalization to a broader population spanning from normal weight to obesity.

## 5. Conclusions

The present analyses suggest a role for salty taste recognition in food reward and uncontrolled eating behavior. Results indicate that a better ability to recognize salty taste may affect implicit motivational components of food reward rather than liking salt and potentially increases awareness of food in the environment, which could be experienced as being controlled by food or losing control over eating behavior, possibly being reflected in trait uncontrolled eating behavior. However, these assumptions and potential underlying mechanisms remain speculative. Nevertheless, brain imaging studies support the assumed associations, demonstrating that brain regions implicated in sensory salt taste processing also modulate reward, executive functioning and executive control in eating in particular [5,6,24,25]. However, these associations do not appear relevant for weight regulation in a cross-sectional approach. Longitudinal studies have to further investigate implications for weight status, food consumption and other metabolic variables. Hypertension may be of high relevance, given that some reports connect dimensions of salty taste perception with hypertension [50]. Due to a lack of evidence that connects salty taste recognition with actual food intake or food selection, further research is needed to untangle the complex interplay of salty taste recognition, food reward and metabolic variables. Addressing salty taste recognition may, nevertheless, be a future target to potentially modify food reward and uncontrolled eating behavior.

## Figures and Tables

**Table 1:** Characteristics of the study population stratified by weight status.

	Sample N = 38	Normal-Weight N = 26 (68.4%)	ObeseN = 12 (31.6%)	*p*-Value *
Body weight (kg)	78.00 (34.03)	68.45 (20.42)	86.75 (38.35)	***p* < 0.001**
Height (cm)	168.00 (15.00)	175.00 (18.00)	169.00 (15.00)	*p* = 0.053
BMI (kg/m^2^)	25.74 (9.78)	22.25 (3.09)	30.71 (15.26)	***p* < 0.001**
Age (years)	33.50 (24.25)	26.00 (27.5)	41.00 (21.50)	*p* = 0.155
Gender N = male/female (%)	13/25 (34.2/65.8)	10/16 (37.5/62.5)	3/9 (27.3/72.7)	^+^ *p* = 0.330
Smoking ^#^ N = yes/no (%).	6/32 (15.80/84.20)	2/14 (12.50/87.50)	4/18 (18.20/81.80)	^+^ ***p* < 0.001**
State hunger (mm) ^ß^	45.00 (50.00)	50.00 (40.00)	35.00 (60.00)	*p* = 0.510
Sweet taste recognition	4.00 (0.00)	4.00 (0.00)	4.00 (0.25)	*p* = 0.804
Salty taste recognition	2.00 (2.00)	2.0 (2.00)	2.00 (2.00)	*p* = 0.651
PFS total	2.43 (1.20)	2.43 (0.95)	2.43 (1.02)	*p* = 0.388
POF availability	1.80 (1.50)	1.83 (0.79)	1.83(1.50)	*p* = 0.492
POF present	2.50 (1.35)	2.00 (1.37)	2.88 (1.31)	*p* = 0.072
POF taste	2.80 (1.25)	2.90 (1,25)	2.70 (1.00)	*p* = 0.372
Uncontrolled eating	4.00 (5.00)	3.00 (2.75)	5.00 (5.25)	*p* = 0.073
Restraint eating	6.00 (6.00)	4.50 (5.75)	6.00 (5.00)	*p* = 0.529
Cognitive restraint	8.00 (6.25)	6.50 (5.75)	10.00 (7.00)	*p* = 0.781
Emotional eating	1.00 (2.00)	0.00 (2.00)	1.00 (2.00)	*p* = 0.298
Hunger	5.00 (4.00)	4.50 (5.00)	5.00 (4.50)	*p* = 0.455
Disinhibition	7.00 (6.00)	5.50 (5.00)	9.00 (4.25)	***p* = 0.013**
EW HFSA	68.40 (35.57)	64.50 (25.68)	71.00 (27.82)	*p* = 0.284
EW LFSA	54.38 (20.75)	53.13 (12.12)	56.38 (34.49)	*p* = 0.529
EW HFSW	41.38 (41.32)	40.0 (36.87)	45.88 (47.82)	*p* = 0.693
EW LFSW	70.00 (24.82)	73.75 (21.13)	64.88 (33.25)	*p* = 0.438
EL HFSA	71.13 (22.50)	67.63 (20.62)	72.88 (26.31)	*p* = 0.510
EL LFSA	56.88 (24.56)	53.13 (21)	65.00 (27.38)	*p* = 0.312
EL HFSW	57.00 (39.65)	56.25 (40.12)	57.00 (41.94)	*p* = 0.693
EL LFSW	71.25 (28.61)	74.75 (25.20)	67.00 (36.00)	*p* = 0.258
IW HFSA	10.97 (39.34)	4.93 (48.05)	24.39 (40.76)	*p* = 0.108
IW LFSA	−17.87 (28.29)	−22.90 (30.78)	−14.25 (30.55)	*p* = 0.421
IW HFSW	−32.97 (57.49)	−34.57 (47.36)	−27.19 (58.18)	*p* = 0.965
IW LFSW	24.82 (48.00)	43.59 (49.47)	18.60 (53.99)	*p* = 0.129

The data are presented as the median (interquartile range). * Mann–Whitney U-test; ^+^ chi-Squared test. *p*-values < 0.05 were considered statistically significant and are highlighted in bold. ^#^ Smoking was defined as smoking at least one cigarette per day, each day of the week. ^ß^ Current hunger was assessed on a 100 mm visual analog scale ranging from 0 = not hungry to 100 mm = very hungry. Abbreviations: BMI, body mass index; IW, implicit wanting; EW, explicit wanting; EL, explicit liking; LFSW, low-fat sweet; LFSA, low-fat savory; HFSW, high-fat sweet; HFSA, high-fat savory.

**Table 2:** Association of sweet and salty taste recognition and LFPQ-G explicit liking and wanting and implicit wanting.

	Explicit Liking				Explicit Wanting				Implicit Wanting			
Taste Quality	HFSA	LFSA	HFSW	LFSW	HFSA	LFSA	HFSW	LFSW	HFSA	LFSA	HFSW	LFSW
**Salty**	r = 0.267 (*p* = 0.105)	r = −0.066 (*p* = 0.695)	r = 0.004 (*p* = 0.980)	r = 0.118 (*p* = 0.481)	r = 0.297 (*p* = 0.070)	r = 0.124 (*p* = 0.458)	r = 0.029 (*p* = 0.865)	r = −0.192 (*p* = 0.249)	r = 0.349 (*p* = 0.032 *)	r = −0.222 (*p* = 0.181)	r = 0.046 (*p* = 0.784)	r = −0.178 (*p* = 0.284)
**Sweet**	r = −0.120 (*p* = 0.473)	r = 0.012 (*p* = 0.944)	r = 0.129 (*p* = 0.440)	r = 0.168 (*p* = 0.313)	r = 0.061 (*p* = 0.715)	r = 0.027 (*p* = 0.872)	r = 0.142 (*p* = 0.395)	r = 0.171 (*p* = 0.305)	r = − 0.195 (*p* = 0.242)	r = − 0.377 (*p* = 0.020 *)	r = 0.160 (*p* = 0.337)	r = 0.244 (*p* = 0.140)

Spearman’s rank correlation coefficient was used to assess associations in 38 participants. *p*-value < 0.05 was considered statistically significant. *p* < 0.05 *. Abbreviations: N, Number; IW, implicit wanting; EW, explicit wanting; EL, explicit liking; LFSW, low-fat sweet; LFSA, low-fat savory; HFSW, high-fat sweet; HFSA, high-fat savory.

**Table 3:** Association of sweet and salty taste recognition and Power of Food Scale.

Taste Quality	Total Score	Availability	Taste	Present
**Salty**	r = 0.371 (*p* = 0.022 *)	r = 0.181 (*p* = 0.276)	r = 0.284 (*p* = 0.084)	r = 0.442 (*p* = 0.005 **)
**Sweet**	r = 0.389 (*p* = 0.016 *)	r = 0.217 (*p* = 0.191)	r = 0.522 (*p* = 0.001 **)	r = 0.194 (*p* = 0.243)

Spearman’s rank correlation coefficient was used to assess associations in 38 participants. *p*-value < 0.05 was considered statistically significant. *p* < 0.05 *, *p* < 0.01 **.

## Data Availability

The original contributions presented in the study are included in the article/Appendix A, further inquiries can be directed to the corresponding authors.

## Abbreviations

LFPQ-G (German Leeds Food Preference Questionnaire); HFSW (high-fat sweet); LFSW (low-fat sweet); HFSA (high-fat savory); LFSA (low-fat savory); BMI (body mass index); IW (implicit wanting); EL (explicit liking); EW (explicit wanting).

## Appendix A

**Table A1:** Association of participants’ characteristics with taste recognition, trait eating behavior and measures of different aspects of hedonic eating.

	Age	Sex	BMI	Smoking ^#^	State Hunger ^ß^
	(r/*p*)	(r/*p*)	(r/*p*)	(r/*p*)	(r/*p*)
**Sweet taste**	−0.458/0.004 **	−0.084/0.617	−0.178/0.286	0.317/0.053	−0.156/0.348
**Salty taste**	−0.097/0.561	0.184/0.268	−0.137/0.414	−0.173/0.299	−0.105/0.532
**Power of Food Scale**					
**Total score**	−0.358/0.027 *	0.091/0.589	0.134/0.422	−0.125/0.453	0.042/0.803
**Availability**	−0.314/0.055	−0.070/0.676	0.194/0.244	−0.218/0.188	0.136/0.416
**Taste**	−0.170/0.308	0.132/0.428	0.224/0.175	−0.056/0.737	−0.068/0.687
**Present**	−0.506/0.001 **	0.137/0.411	−0.186/0.263	0.060/0.723	0.070/0.678
**Leeds Food Preference Qestionnaire-G**					
**EL (HFSA)**	0.105/0.531	−0.075/0.655	0.144/0495	−0.372/0.022 *	−0.051/0.760
**EL (LFSA)**	−0.136/0.417	0.142/0.395	0.122/0.466	−0.138/0.408	−0.003/0.985
**EL (HFSW)**	−0.298/0.070	−0.046/0.782	0.117/0.483	−0.020/0.906	0.207/0.213
**EL (LFSW)**	−0.175/0.294	−0.139/0.404	−0.166/0.318	0.269/0.071	0.101/0.548
**EW (HFSA)**	0.133/0.499	−0.096/0.568	0.154/0.357	−0.395/0.014 *	−0.015/0.929
**EW (LFSA)**	0.082/0.624	0.046/0.782	0.119/0.475	−0.211/0.204	0.080/0.634
**EW (HFSW)**	−0.166/0.319	−0.005/0.975	0.124/0.458	−0.023/0.891	0.275/0.094
**EW (LFSW)**	−0.128/0.444	−0.067/0.689	−0.128/0.444	0.306/0.062	0.096/0.565
**IW (HFSA)**	0.268/0.104	0.021/0.902	0.181/0.277	−0.322/0.048 *	−0.120/0.474
**IW (LFSA)**	0.474/0.003 **	0.227/0.170	0.201/0.226	−0.125/0.454	−0.149/0.373
**IW (HFSW)**	−0.241/0.145	−0.077/0.644	0.073/0.665	−0.007/0.969	0.057/0.733
**IW (LFSW)**	−0.339/0.037 *	−0.062/0.712	−0.391/0.015 *	0.467/0.003 **	0.157/0.346
**Three-Factor Eating Questionnaire**					
**Disinhibition**	0.007/0.966	−0.230/0.165	0.464/0.003 **	−0.160/0.337	−0.139/0.404
**Hunger**	−0.194/0.234	−0.138/0.410	0.116/0.488	0.023/0.890	0.112/0.503
**Restraint eating**	0.190/0.253	0.078/0.643	0.384/0.017 *	−0.122/0.467	−0.019/0.908
**Cognitive restraint**	0.164/0.326	0.128/0.442	0.264/0.109	−0.048/0.776	0.021/0.902
**Emotional eating**	−0.024/0.886	−0.489/0.002 **	0.236/0.155	−0.266/0.106	0.099/0.553
**Uncontrolled eating**	−0.144/0.387	−0.068/0.685	0.199/0.231	−0.031/0.851	−0.193/0.245

Spearman’s rank correlation coefficient was used to assess associations in 38 participants. *p*-value < 0.05 was considered statistically significant and given as *p* < 0.05 *, *p* < 0.01 **. ^#^ Smoking was defined as smoking at least one cigarette per day, each day of the week. ^ß^ State hunger was assessed on a 100 mm visual analog scale ranging from 0 = not hungry to 100 mm = very hungry. Abbreviations: IW, implicit wanting; EW, explicit wanting; EL, explicit liking; LFSW, low-fat sweet; LFSA, low-fat savory; HFSW, high-fat sweet; HFSA, high-fat savory.
